# The balancing act between lipid droplets and lysosomes for membrane functionality in age-related neurodegeneration and inflammation

**DOI:** 10.1016/j.plipres.2025.101341

**Published:** 2025-06-05

**Authors:** Mariana I. Tsap, Halyna R. Shcherbata

**Affiliations:** a Institute of Cell Biochemistry, Hannover Medical School, Carl-Neuberg-Strasse 1, 30625 Hannover, Germany; b Mount Desert Island Biological Laboratory, Bar Harbor, ME 04609, USA

**Keywords:** Lipid droplets, Fatty acids, PNPLA6/NTE, Inflammaging, Lipid membranes, Lysosomes

## Abstract

Age-related neurodegenerative disorders are often associated with disruptions in lipid metabolism. A critical aspect is the impairment of the interaction between lipid droplets (LDs) and lysosomal function, leading to the accumulation of toxic lipid species. This accumulation triggers cellular stress, inflammation, and defective waste processing within cells, disrupting cellular homeostasis and amplifying neuroinflammatory processes. Recent studies have shown that alterations in phospholipid and fatty acid homeostasis drive neuroinflammation and oxidative stress, exacerbating neurodegenerative processes. This review focuses on the role of neuropathy target esterase (PNPLA6/NTE) and NTE-related esterase (PNPLA7/NRE) in lipid metabolism, highlighting how dysregulation of these enzymes contributes to neurodegeneration, inflammation, and lysosomal dysfunction. Additionally, we discuss the involvement of lipid rafts, sphingolipids, and phospholipase enzymes, particularly PLA2 family members, in cellular signaling and membrane dynamics. By examining the relationship between lipid metabolism, inflammatory signaling, and lysosomal storage disorders, we aim to provide a comprehensive understanding of how LDs and lysosomes interact to influence cellular homeostasis in neurodegenerative conditions, which could lead to new therapeutic strategies addressing lipid dysregulation in age-related neurological disorders.

## 1. The global burden of neurological disorders and inflammaging

**Fig. 1.**
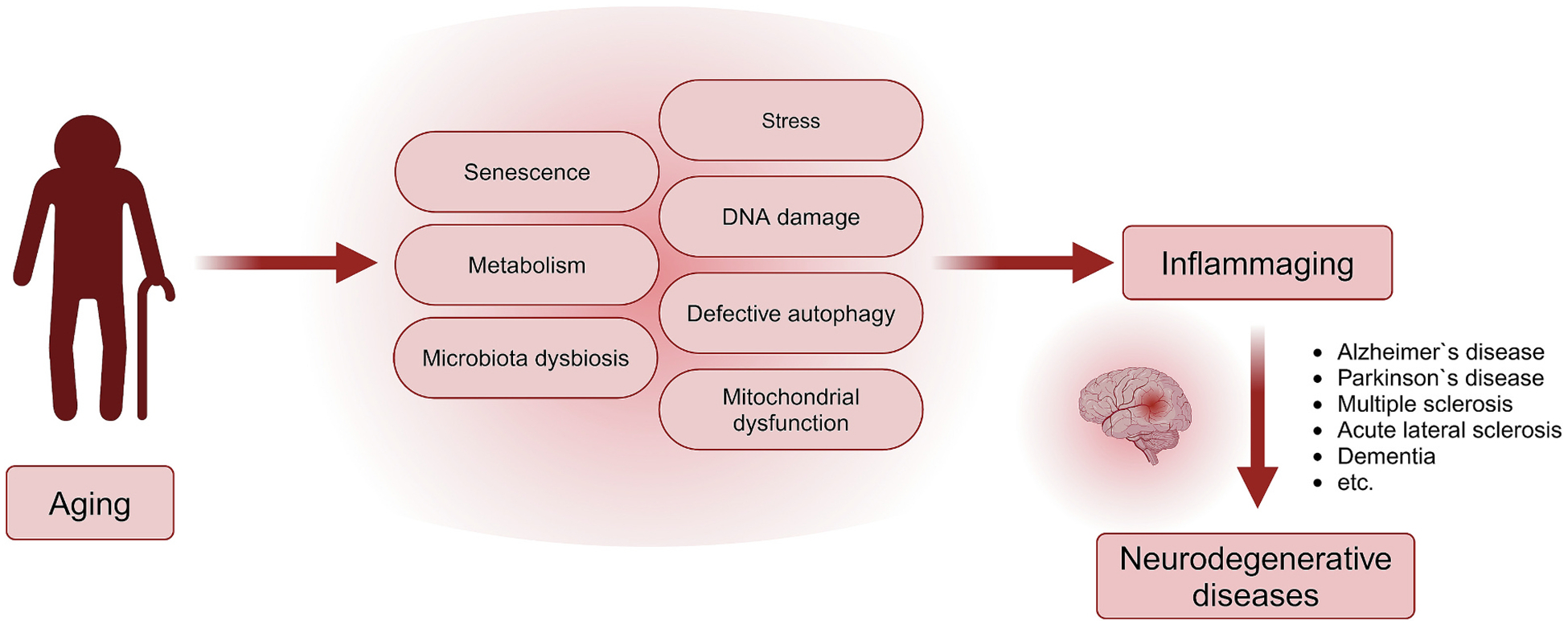
The global burden of neurological disorders and inflammaging. The rising incidence of neurological disorders can be attributed to multiple factors, including global population growth and aging, as well as increased exposure to environmental, metabolic, and lifestyle-related risks. Key contributors include stress, microbiome alterations, metabolic imbalances, DNA damage, mitochondrial dysfunction, and impaired autophagy. These factors drive inflammaging, a chronic, aging-related inflammatory process that plays a crucial role in the progression of neurological disorders such as Alzheimer’s disease, Parkinson’s disease, and multiple sclerosis. Created in BioRender. Tsap, M. (2025) https://BioRender.com/b74p228

Affecting 43 % of the world’s population, neurological conditions are the leading cause of mortality and disability combined, and second as a cause of global deaths. With 9 million deaths annually and substantial economic costs, addressing this crisis has become a priority, highlighted by WHO’s Intersectoral Global Action Plan on Epilepsy and Other Neurological Disorders 2022–2031 (IGAP) [1]. Over the last three decades, there has been a significant global increase in the prevalence of neurological conditions like stroke, Alzheimer’s disease, other dementias, and meningitis. This rise can be attributed to factors such as the expansion and aging of the global population, along with heightened exposure to environmental, metabolic, and lifestyle-related risk factors [2,3]. The aging process and age-related diseases are interconnected through fundamental mechanisms, mostly centered on inflammation (Fig. 1). As individuals age, there is a development of chronic, sterile, low-grade inflammation known as inflammaging, playing a role in the onset and progression of age-related diseases [4]. Neuroinflammation is crucial in the progression of diverse neurological disorders, including immunological conditions such as multiple sclerosis (MS) but also neurodegenerative diseases like Alzheimer’s disease (AD) and Parkinson’s disease (PD), along with brain injuries such as stroke and traumatic brain injury (TBI) [5]. Therapeutic strategies targeting neuroinflammation offer innovative approaches to modulate the immune response in order to alleviate neurodegenerative processes [5].

## 2. Lipid metabolism and neuroinflammation: Insights into microglia, lipid rafts, and lipid droplets in neurodegenerative diseases

Lipids are fundamental to cellular structure and function, serving roles in stabilizing cells and transmitting signals. Variations in lipid composition across cell types and organelles highlight their diverse functional requirements. Proper lipid organization within cellular compartments is crucial for regulating processes like signal transduction and membrane dynamics. For example, cholesterol plays a critical role in organizing lipid structures, with tightly regulated mechanisms maintaining cellular cholesterol levels to facilitate proper trafficking, while its imbalance leads to disorders, such as in Niemann–Pick type C or atherosclerosis. Additionally, alterations in choline phospholipids levels, such as lysophosphatidylcholine (LPC) [6], and sphingolipids, including sphingosine, ceramide 1-phosphate, and ceramide can contribute to pathological conditions such as AD [7,8]. Changes in lipid composition and metabolism have been identified in the context of inflammaging. The complex interplay between the lipid alterations and their effects on inflammatory signaling pathways creates a multifaceted relationship, warranting further exploration and analysis. This is especially important for understanding the potential implications for the development and progression of inflammaging and neurodegenerative diseases [9,10].

For example, the bioactive lysophospholipids—particularly lysophosphatidic acid (LPA) and sphingosine-1-phosphate (S1P)—act as ligands for G protein-coupled receptors (GPCRs) and mediate a wide range of cellular responses. Moreover, receptors for both LPA and S1P play key roles in inflammatory processes, including neuroinflammation, and are implicated in various neuropathological conditions such as MS, HD, amyotrophic lateral sclerosis (ALS), stroke, and AD [11–15].

Phosphoinositides, a group of signaling lipids highly enriched in the brain, are distributed across distinct cellular membranes. Interestingly, phosphatidylinositol 4,5-bisphosphate (PI(4,5)P_2_) has been identified as a critical regulator of Toll-like receptor 4 (TLR4) signaling, a pathway vital for maintaining nervous system integrity [16–18].

Microglia’s lipid metabolism, involving saturated and polyunsaturated fatty acids (PUFAs), and lysophospholipids, has been increasingly linked to changes in brain function, with dysregulation in conditions such as obesity, aging, and neurodegenerative diseases like AD exacerbating neuroinflammation and promoting the accumulation of toxic proteins, thereby driving disease progression [19]. Microglia, the central nervous system’s resident immune cells, is essential for maintaining brain homeostasis and defending against inflammation, while microglia dysregulation can lead to chronic inflammatory responses contributing to neuroinflammation in AD and PD [20–24].

Endocannabinoids play regulate sleep, mood, appetite, memory, and play crucial roles as lipid signaling molecules in various physiological processes, especially in the brain. Anandamide (AEA) and 2-arachidonoyl glycerol (2-AG) are the most studied among them. Their dysregulation has been linked to neurodegenerative conditions, highlighting their significance in neurological health [25,26]. Endocannabinoids act through cannabinoid receptor type 1 (CB1) and type 2 (CB2). Activation of CB2 receptors has been shown to promote the clearance of native amyloid-beta (Aβ) from human frozen brain tissue sections [27]. Meanwhile, selective activation of CB1 receptors can inhibit nitric oxide (NO)-dependent hyperphosphorylation of tau protein in co-cultured PC12 neurons, highlighting the therapeutic potential of the endocannabinoid system in neurodegenerative conditions [28].

Apolipoprotein E (ApoE) serves as the main cholesterol carrier and is a significant genetic risk factor for late-onset AD. ApoE can aggregate within the *endo*-lysosomal system of microglia, a process influenced by immune and lipid metabolism. In AD, increased ApoE expression may promote lipoprotein uptake within microglia, triggering a pathological cascade that leads to Aβ amyloidosis [29,30].

Cholesterol homeostasis and immune responses are tightly intertwined, with intracellular cholesterol distribution being crucial for inflammatory functions. Proper cholesterol trafficking to the endoplasmic reticulum (ER) is necessary for the activation of Nuclear factor-κB (NF-κB) and MAPK signaling, enhancing the expression of pro-inflammatory cytokines like interleukin 6 (IL-6) and tumor necrosis factor α (TNF-α), while also affecting the localization and regulation of pattern-recognition receptors (PRRs) in membrane domains, also known as lipid rafts [31].

Dynamic, cholesterol- and sphingolipid-enriched membrane nano-domains with the potential to form larger microscopic domains are defined as lipid rafts or membrane domains [32,33]. Moreover, rafts mediate specific cellular functions, including cell trafficking, pathogen entry, and cell adhesion. Rafts, particularly membrane cholesterol, facilitate and stabilize the interaction and assembly of specific sets of proteins, for example the integrity of tight junctions (TJs) in processes like blood-brain barrier (BBB) formation [34]. Interestingly, lipid rafts, which are abundant on mature lysosomes, facilitate the transport of AD-related proteins to the lysosomal system. This process affects amyloid precursor protein (APP) processing and is a key component of the autophagic-lysosomal pathway. Evidence of this includes the presence of gamma-secretase subunits in lysosomes, which interact with Aβ peptides and cholesterol. The lysosomal membrane, derived from various subcellular pathways, is influenced by these lipid rafts, impacting APP processing and contributing to protein accumulation in AD [35,36].

Another important class of lipids, such as sphingolipids, act not only as fundamental components of eukaryotic cell membranes but also as key signaling molecules. They are involved in regulating numerous cellular functions, such as cell growth, division, survival, immune cell migration, maintenance of vascular and epithelial barriers, and inflammatory responses [37–40]. In the brain, sphingolipids are crucial for regulating immune and inflammatory responses by influencing cytokine signaling, eicosanoid production, astroglial activation, T-cell migration, and activating receptor-mediated pathways. Changes in sphingolipid metabolism have been linked to neuronal loss, activation of glial cells, and heightened production of inflammatory mediators. In particular, ceramide has been shown to promote the release of proinflammatory cytokines such as TNF-α, IL-1β, and IL-6, as well as activate the NF-κB [38]. Moreover, elevated ceramide levels can lead to the generation of reactive oxygen species (ROS), mitochondrial dysfunction, and neuronal apoptosis [41].

Other dynamic cellular structures that are involved in diverse cellular processes, degradation, and microbial pathogenesis are LDs. LDs, previously considered simple lipid storage particles, are now recognized as highly regulated organelles with diverse roles in energy storage, signaling precursors, cell stress management, and protein handling. LDs start forming between ER bilayers, utilizing glycerolphosphate and fatty acyl-CoA to synthesize glycerolipids. LD growth involves the transfer of TAGs to LDs through membrane bridges or fusion of two LDs. LDs are surrounded by a polar lipid monolayer with decorating proteins, including perilipins (PLINs), which contribute to LD formation and protect them from lipolysis. LD decomposition occurs through lipolysis or lipophagy, where LD-associated lipases break down triacylglycerols (TAGs) to free fatty acids (FFAs). Mammalian cells have been observed to engage in interactions between LDs and lysosomes. LDs are transported to lysosomes for degradation by lysosomal acid lipase (LAL), facilitated by Rab7. Lipophagy, a form of selective autophagy and a recent focus in lipid metabolism research, refers to the autophagic degradation of intracellular LDs [42]. Several human pathologies have been associated with the dysregulation of the LD life cycle and physiological functions, neutral lipid storage disease, lipodystrophy, and hereditary spastic paraplegia [43]. Lipid storage diseases, a cluster of inherited metabolic disorders, also involve mutations that disrupt lysosomal function, potentially resulting in lysosomal storage disorders (LSDs) [44]. Pathological factors, including neuroinflammation, oxidative stress, and aging, contribute to neuroglial lipid storage. Neuroinflammation triggers the formation of LDs in microglia, potentially promoting age-related inflammation. ROS play a role in both driving and being driven by LD accumulation, creating a destructive cycle. Aging is associated with increased LD accumulation in the brain, possibly linked to age-related neuroinflammation and metabolic changes. LDs in glial cells, especially in microglia and astrocytes, have been associated with neurodegeneration [45–47]. However, the exact role of LDs in the brain, whether protective or detrimental, remains a topic of debate.

LDs regulate cellular FFA levels, contributing to lipotoxicity characterized by ER stress, oxidative damage, and accumulation of bioactive lipid intermediates like diacylglycerol (DAG) and ceramides, which can lead to cell death [48]. Moreover, LDs play a crucial role in maintaining a balance between sequestering and releasing PUFAs. Oxylipins including eicosanoids, bioactive signaling lipids, are derived from arachidonic acid (AA) and other PUFAs, and regulate various homeostatic and inflammatory processes associated with numerous diseases. They play crucial regulatory roles in the brain and throughout the rest of the body, especially in immune and inflammatory responses. Furthermore, eicosanoids participate in the modulation of immunopathological processes and long-term tissue remodeling [49]. Phospholipase A2 (PLA2) enzymes play a crucial role in elevating PUFAs levels for metabolism and eicosanoid biosynthesis under most physiological conditions [50]. Moreover, there is suggestive associations between specific PLA2 subgroups (Lipoprotein-associated Phospholipase A2 (LPLA2), α/β-hydrolase domain-containing (ABHD), secreted phospholipase A2 (sPLA2), and calcium-independent phospholipase A2 (iPLA2)) and the risk of neurodegeneration [51–53]. In addition, it has been also discovered the association several genes (*PLA2G2D*, *PLA2G12A, PLA2G7, PLA2G15*, and *ABHD12*) and neurological conditions, including schizophrenia, hereditary spastic paraplegia, PHARC (Polyneuropathy, Hearing loss, Ataxia, Retinitis pigmentosa, and Cataract), PD, etc. [54–58]. All the listed lipases belong to the PLA2 family, which is important to study due to its crucial role in lipid metabolism, membrane remodeling, signaling, and its involvement in various diseases, making it significant for both health and disease.

## 3. Exploring Patatin-like phospholipase domain-containing proteins (PNPLAs): Roles in lipid metabolism, disease pathology, and therapeutic potential

Patatin-like phospholipase domain-containing proteins (PNPLAs) belong to the PLA2 superfamily of enzymes or lipases. PNPLAs, characterized by a “patatin domain” initially identified in a potato tuber protein, are crucial for lipid metabolism and signaling. In humans, there are nine PNPLAs, most of which are ubiquitously expressed and found in the cytosol, organelle membranes or cytoplasmic LDs. These proteins hydrolyze lipid substrates, with PNPLA1–5 targeting TAGs and retinylesters (REs), and acting as lipases or acyltransferases/transacylases, while PNPLA6–9 primarily hydrolyze phospholipids without acyltransferase activities [59]. Their physiological roles are not fully understood, but it is known that abnormalities in certain PNPLAs are linked to various conditions and diseases (Table 1). For example, PNPLA1 mutations cause autosomal recessive congenital ichthyosis [60], while PNPLA2 mutations can lead to neutral lipid storage disorder characterized by triacylglycerol accumulation in almost every tissue, and myopathy [61,62]. PNPLA3 mutations are associated with several liver diseases and obesity [63,64], and mutations or irregular activity in PNPLA6, PNPLA8 and PNPLA9 are linked to neurological disorders including infantile neuroaxonal dystrophy, atypical neuroaxonal dystrophy, parkinsonian syndrome, spastic paraplegia type 39 (SPG39), Gordon-Holmes syndrome, neurodegeneration, and etc. [65–67]. PNPLAs are involved in various biological processes, such as regulating energy metabolism, maintaining membrane homeostasis, and facilitating cell signaling. Although not all PNPLA enzymes are expressed in the nervous system or directly linked to neurodegenerative disorders, their regulatory mechanisms share significant similarities that may provide insight into their molecular functions. For instance, several PNPLAs, including PNPLA1–3, have been shown to interact with ABHD5/CGI-58, which regulates their localization and activity. PNPLA7 and PNPLA8 both play roles in hepatic phosphatidylcholine (PC) catabolism, while PNPLA7 and PNPLA6 share structural similarities. Additionally, PNPLA8 and PNPLA6 are implicated in maintaining nervous system integrity. Furthermore, PNPLA2, PNPLA8, and PNPLA9 are associated with AA metabolism, contributing to the regulation of inflammatory mediators and activation of inflammatory responses.

PNPLA1 is involved in the synthesis of omega-O-acylceramides by transferring linoleic acid from TAG to an omega-hydroxyceramide. PNPLA1 is expressed in differentiated keratinocytes and is crucial for the skin barrier [68,69]. In cells, PNPLA1 is a free enzyme in the cytoplasm but can also bind to LDs. The pathology of Autosomal Recessive Congenital Ichthyosis (ARCI), a rare genetic disease with lifelong barrier dysfunction and generalized scaly and inflamed skin, associated with PNPLA1 mutation is caused by reduced omega-acylceramide levels [70]. PNPLA1 global or keratinocyte-specific loss impairs epidermal barrier formation by disrupting acylceramide synthesis, leading to neonatal lethality, altered keratinocyte differentiation, and ichthyosis-like symptoms, which can be partially rescued by acylceramide supplementation [68]. Moreover, PNPLA1 mutations (p.D172N and p. Y245del) in ARCI patients cause abnormal LD accumulation in fibroblast cells, disrupting lipophagy and the degradation of these droplets [71,72]. ABHD5/CGI-58 promotes PNPLA1-mediated acylceramide synthesis, indicating that ABHD5/CGI-58 functions to retain TAGs in the ER, the site of acylceramide production, and facilitates their presentation to PNPLA1 for effective substrate recognition. ABHD5/CGI-58 facilitates the recruitment of PNPLA1 to the LD membrane. When both proteins are highly expressed, LDs disappear. In cells with low PNPLA1 and ABHD5/CGI-58 expression, association of PNPLA1 with LD membranes reflects an intermediate step that anticipates the droplet loss seen under higher expression conditions [73].

PNPLA2 encodes adipose triglyceride lipase (ATGL), which catalyzes the hydrolysis of TAG at the sn-2 (or sn-1) positions on the surface of LDs, initiating TAG degradation during fasting to obtain energy, a process known as lipolysis [74]. It was shown that PNPLA2 activity is regulating by G0/G1 Switch 2 (G0S2) that localizes to LDs and inhibits their PNPLA2-mediated degradation by directly binding to PNPLA2 via its hydrophobic domain [75]. Mutations in this gene are linked to neutral lipid storage disease with myopathy [62]. PNPLA2 deficiency results in defective lipolysis, accompanied by increased glucose tolerance, insulin sensitivity, and increased the respiratory quotient during fasting, suggesting that reduced FFA availability shifts metabolism toward glucose use, even with excess fat in tissues [76]. Moreover, activation of cardiac and hepatic gene transcription by the PPAR-α–PGC-1 complex relies on PNPLA2-mediated lipolysis of cellular TAGs, which produces key lipid ligands necessary for PPAR activation [77]. Moreover, loss of PNPLA2 or hormone-sensitive lipase (Hsl), which inhibits lipolysis, alleviates specific aspects of cancer-associated cachexia (CAC) [78]. Interestingly, PNPLA2 regulates cancer cell proliferation via AMP-kinase and mTOR signaling, while enhancing the lipolytic pathway [79,80]. During nutrient deprivation, LDs can release fatty acids through lipolysis or lipophagy, which mitochondria then use for energy production via β-oxidation and the citric acid cycle [43]. The trafficking of these fatty acids probably happens at contact sites. The perilipin family protein PLIN5 controls lipolysis by interacting with PNPLA2. PNPLA2 regulates inflammatory response [81], and cancer cell proliferation through mTOR signaling, while also boosting the lipolytic pathway [79,80]. In addition, ABHD5/CGI-58 regulates PNPLA2 activity on adipocyte LDs through phosphorylation-dependent interactions with PLIN1, thereby activating PNPLA2-mediated lipolysis of cellular fat stores—a process disrupted in Chanarin-Dorfman Syndrome [82–84]. A recent study reported that PNPLA2 uses its transacylase activity to synthesize fatty acid esters of hydroxy fatty acids, which have potential anti-inflammatory roles [81]. Moreover, exogenous AA or oleic acid induces LD formation and PNPLA2- and cPLA2-dependent prostaglandin I2 (PGI2) release. PNPLA2-dependent lipolysis regulates endothelial barrier function and maintains vascular homeostasis by producing PGI2 from endogenous AA, suggesting that lipid overload in the vascular wall or endothelial/smooth muscle cells triggers LD formation, activating the PNPLA2-cPLA2-AA-PGI2 pathway to protect endothelial function [85]. PNPLA2 regulates the release of PUFAs from LD stores and their conversion into cyclooxygenase- and lipoxygenase-derived lipid mediators under both nutrient-rich and serum-starved conditions; during starvation, it also facilitates the incorporation of LD-derived PUFAs into phospholipids, providing substrates for cPLA2α [86].

Similar to PNPLA2, PNPLA3 is found in the cytosol or bound to LDs. The PNPLA3 (or adiponutrin) gene encodes a lipase with acyltransferase activity, primarily expressed in the liver, especially in hepatocytes and hepatic stellate cells. PNPLA3 functions as a TAG hydrolase at the sn-2 position of the glycerol backbone and also acts as a thioesterase and acyltransferase on phospholipids [87,88]. Moreover, PNPLA3 mobilizes polyunsaturated fatty acids to support the hepatic secretion of large very low-density lipoprotein particles [89]. Mutations in PNPLA3 lead to elevated triglyceride levels and are strongly associated with the heritability of metabolic dysfunction-associated fatty liver disease (MAFLD), metabolic dysfunction-associated steatohepatitis (MASH) and other liver diseases, increasing susceptibility to fatty liver diseases and complications like hepatocellular carcinoma (HCC), closely tied to chronic inflammation [90]. In mice, the PNPLA3 I148M variant leads to its accumulation on LDs due to impaired protein turnover, as the mutant form escapes ubiquitylation and subsequent proteasomal degradation, interfering with TAG hydrolysis by displacing PNPLA2 or its cofactor, ABHD5/CGI-58, thereby inhibiting PNPLA2/ATGL-mediated lipolysis [91]. Moreover, recent study revealed that the PNPLA3 I148M carriers are characterized by hepatic mitochondrial dysfunction. The PNPLA3 I148M variant shows elevated β-oxidation and ketogenesis, possibly due to increased fatty acid entry into mitochondria, leading to increased mitochondrial redox state, suggesting that reduced de novo lipogenesis could contribute to the progression from hepatic steatosis to advanced liver disease and fibrosis upon pathological conditions [92].

PNPLA4 (or GS2, iPLAη), the smallest member of the PNPLA family, is able to hydrolyze retinylesters (REs), esterifying retinol using various acyl donors. PNPLA4 is expressed in nearly all tissues of the body, including adipose tissue, liver, muscles, kidneys, lungs, placenta, and brain [93,94]. PNPLA4 is expressed across diverse species, from primates and other mammals to birds, reptiles, amphibians, and fish; however, mice appear to lack a PNPLA4 homolog based on genomic data and published reports [95]. Loss of PNPLA4 in the immortalized epithelial cell line SCC12b promotes RE accumulation within the cell, potentially functioning as either a catalyst or a regulatory protein enhancing RE formation catalyzed by other acyl transferases [96].

Although PNPLA5 (or GS2-like) is ubiquitously expressed in low levels across most tissues, its expression patterns vary among species. PNPLA5 exhibits TAG hydrolase activity. Whole-exome sequencing in human revealed that PNPLA5 is associated with low density lipoprotein cholesterol (LDL-C) [97]. Recent study revealed that PNPLA5-knockout induced apoptosis in rat testes, as well as it influences the expression level of proteins involved in steroid metabolism and wound healing process [98]. Furthermore, PNPLA5, found within LDs, appeared to be essential for the efficient initiation of autophagy, facilitating the degradation of various substrates such as autophagic adaptors, bulk proteolysis, regulation of mitochondrial quantity, and microbial clearance [99].

PNPLA8 (or iPLA2γ, iPLA2–2) is highly expressed in the myocardium and, to a lesser extent, in the placenta, skeletal muscles, brain, liver, pancreas, and lungs [100]. PNPLA8 can be localized to peroxisome and mitochondrial membranes, as well as to the ER and autophagosomes. PNPLA8 displays PLA2 activity toward phospholipids bearing sn-2 saturated or monounsaturated fatty acid, while it acts as a PLA1 toward phospholipids bearing sn-2 PUFA. This unique feature of PNPLA8 identifies a novel metabolic pathway that generates 2-arachidonoyl-LPC (2-AA-LPC), a crucial metabolite in several signaling pathways, including inflammatory response [101]. Moreover, PNPLA8 activity leads to the release of AA, production of 2-AA-LPC, and generation of eicosanoid metabolites. These studies also demonstrate decreased eicosanoid production in mitochondria upon PNPLA8 loss, highlighting that PNPLA8, regulated by Ca2+ and Mg2+ ions, orchestrates cellular bioenergetic and signaling responses through the coordinated release of AA and production of downstream metabolites [102]. Additionally, PNPLA8 hydrolyzes cardiolipin, a phospholipid found exclusively in the mitochondrial membrane and essential for normal mitochondrial function [103,104]. PNPLA8 was shown to play a role in the insulin release and insulin sensitivity [105]. It was shown that PNPLA8 and PNPLA7 act as key players in hepatic PC catabolism for endogenous generation of glycerophosphocholine (GPC) and choline, whose methyl groups are preferentially utilized in the methionine cycle. Genetic deficiency of either PNPLA7 or PNPLA8 perturbs hepatic PC catabolism, resulting in phenotypes reminiscent of methionine insufficiency [106]. Another study demonstrated that PNPLA8 overexpression markedly reduces hepatic steatosis in high-fat diet (HFD)-fed mice by enhancing autophagy in hepatocytes [107]. Moreover, PNPLA8 loss leads to abnormal mitochondrial function, ROS generation, elevated lipid peroxidation, and ultimately apoptosis [108], as well as muscle atrophy [109]. Interestingly, loss of PNPLA8 has been reported to be associated with the progressive neurodegenerative phenotypes, such as microcephaly, cerebellar ataxia and peripheral neuropathy [67,110,111].

PNPLA9 (or PLA2G6, iPLA2β, GVIA iPLA2) contains ankyrin repeats and a calmodulin-binding domain for protein interactions. Its crystal structure reveals that it forms a stable dimer in its active form [65]. PNPLA9 interacts with ATP, Ca2+/calmodulin-dependent protein kinase II (CaM kinase), and the chaperone protein calnexin. PNPLA9 is primarily expressed in the cytoplasm [112] where it binds to various organelles such as the cell membrane, mitochondria, endoplasmic reticulum, Golgi apparatus, and nuclear envelope [87,113–116]. The PNPLA9 protein is widely expressed in the tissues of various animal species including humans and mice. Mutations in PNPLA9 are linked to various neurodegenerative diseases, including infantile neuroaxonal dystrophy, atypical neuroaxonal dystrophy, and a parkinsonian syndrome characterized by adult-onset dystonia and autosomal recessive early-onset parkinsonism [117–119]. Interestingly, PNPLA9 activity was increased in patients with psychosis and schizophrenia [120,121]. PNPLA9 plays a role in inflammation, immune responses, cell proliferation, apoptosis and remodeling of membrane phospholipids [122,123]. PNPLA9 can preferentially hydrolyze peroxidized phospholipids. In addition, loss of PNPLA9 leads to upregulated mitochondrial lipid peroxidation and mitochondrial dysfunction [124]. Furthermore, PNPLA9 prevents ferroptosis, that is caused by the accumulation of 15-hydroperoxy (Hp)-arachidonoyl-phosphatidylethanolamine (15-HpETE-PE), produced by complexes involving 15-lipoxygenase (15-LOX) and the scaffold protein phosphatidylethanolamine (PE)-binding protein (PEBP) 1. Interestingly, PNPLA9 deficiency leads to the accumulation of 15-HpETE-PE, contributing to progressive parkinsonian motor deficits [125,126]. Moreover, PNPLA9 is a critical regulator for p53-driven ferroptosis ROS-induced stress [127]. Interestingly, PNPLA9 acts as a “cellular compass,” influencing monocyte migration in response to the chemokine monocyte chemoattractant protein (MCP-1/CCL2). CCL2 crucial in inflammatory disorders, stimulates chemotaxis, directing monocytes toward inflamed sites [128,129]. Dysfunction of PNPLA9-associated AA metabolism activates inflammatory responses through inflammatory mediator regulation [130]. In addition, the PNPLA9-null mice showed protection against diet-induced body and liver weight gain, liver enzyme elevation, serum free fatty acids, and hepatic triglycerides, as well as reduced steatosis scores. PNPLA9 deficiency improved phospholipid remodeling by reducing levels of LPC, lysophosphatidylethanolamine (LPE), and lysophosphatidylinositol (LPI), while increasing hepatic arachidonate and arachidonate-containing cholesterol esters and PGE2 [131]. Although many functions of PNPLA enzymes and their underlying mechanisms have been uncovered, there are still some gaps in our understanding of disease pathology.

This table highlights the characteristics of PNPLA (patatin-like phospholipase domain-containing protein) family members, including their alternative names, subcellular localization, substrates, tissue distribution, primary functions, and associated diseases.

## 4. PNPLA6/NTE and PNPLA7/NRE in lipid metabolism, neurodevelopment, and disease pathology

**Fig. 2.**
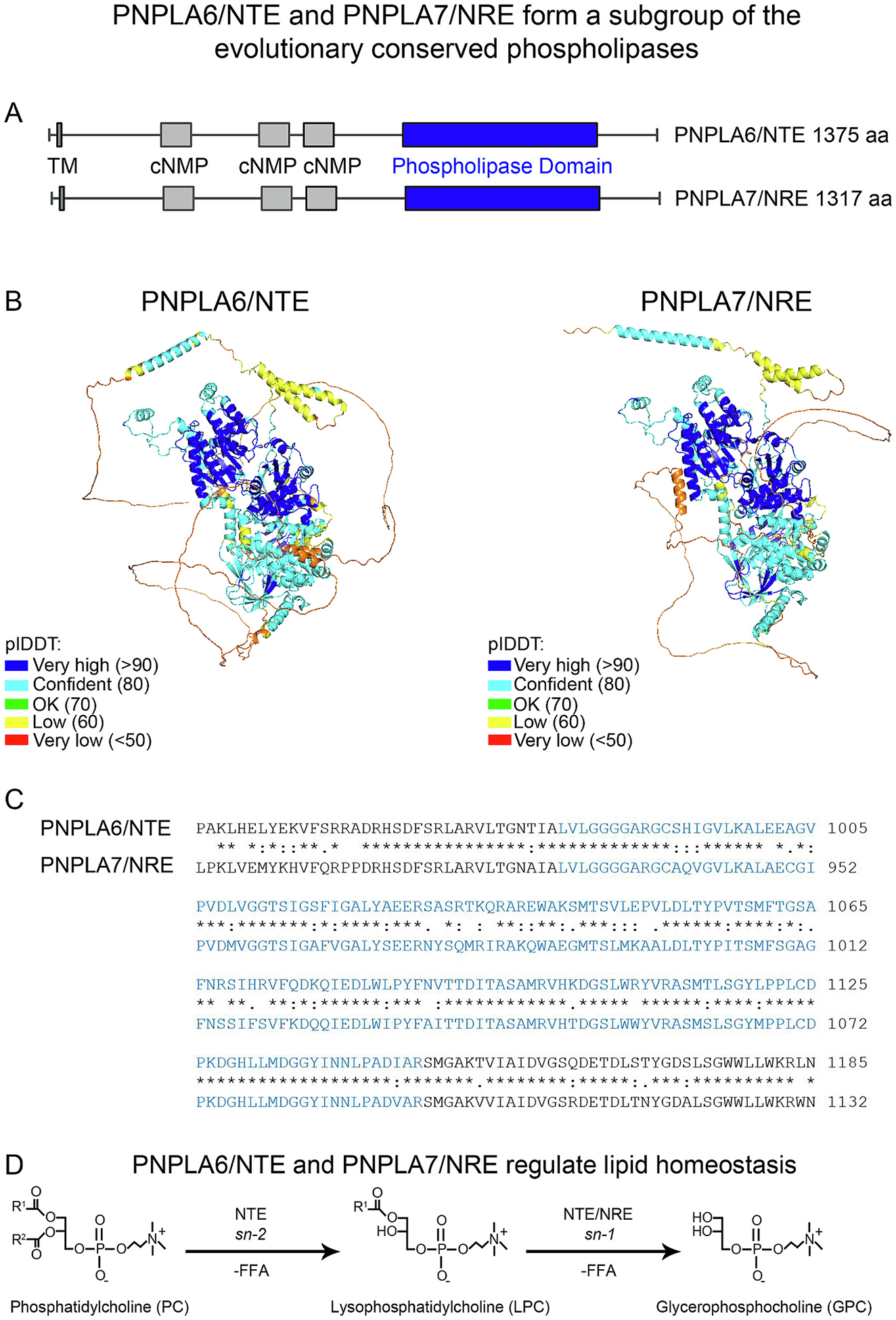
PNPLA6/NTE and PNPLA7/NRE form a subgroup of the evolutionary conserved phospholipases. A. Schematic of human PNPLA6/NTE (1375 aa) and PNPLA7/NRE (1317 aa). The following domains are displayed: TM is represented as a grey vertical line, cNMP as grey boxes, and the phospholipase domain as a blue box [166]. B. The 3D structures of human PNPLA6/NTE and PNPLA7/NRE proteins were generated using AlphaFold3 and visualized with PyMOL. Both proteins feature a highly conserved patatin-like phospholipase domain (pLDDT>90), signifying high accuracy and reliability in their structural predictions. C. Aligned sequences of the conserved patatin-like phospholipase domain in PNPLA6/NTE (residues 981–1147) and PNPLA7/NRE (residues 928–1094). D. PNPLA6/NTE hydrolyzes PC by first cleaving the sn-2 position, resulting in the formation of LPC and the release of a FFA. Both PNPLA6/NTE and PNPLA7/NRE can subsequently hydrolyze the sn-1 position, producing GPC and an additional FFA. R1 and R2 represent the hydrocarbon chains of the FFA components within the phospholipid structure [66].

PNPLA6/NTE and PNPLA7/NRE feature a unique domain architecture compared to other PNPLAs, and the NTE family has an evolutionary origin distinct from the other two PNPLA subgroups (either the adiponutrin (ADPN) group (PNPLA1–5) or PNPLA8–9 group) [132,133]. PNPLA7/NRE shows high homology to PNPLA6/NTE, with 73 % homology in mice and 74 % in humans (61 % identity in both). Both proteins possess a highly conserved domain architecture consisting of the enzymatic patatin-like phospholipase domain, along with transmembrane domain (TM), and with extensive “non-enzymatic” segments whose functions remain poorly defined, including three putative cyclic nucleotide monophosphate (cNMP)-binding sites [134]. The PNPLA6/PNPLA7 genes are highly evolutionarily conserved (Fig. 2), with orthologues found in diverse species such as *Drosophila*, zebrafish, chicken, mouse, rat, and chimpanzee [66].

PNPLA6/NTE, previously known as neurotoxic esterase [135], is primarily recognized for its role in organophosphate-induced delayed neuropathy (OPIDN), a condition caused by systemic inhibition of PNPLA6/NTE by specific organophosphorus (OP), found in a variety of commercial products such as flame retardants, insecticides, pesticides, and pharmaceuticals. OPIDN is characterized by axonopathy resulting in the degeneration of peripheral and central motor and sensory neurons; however, it also plays a significant role in neuronal development and brain function [66].

PNPLA6/NTE demonstrates ubiquitous expression across a diverse array of tissues in humans https://www.proteinatlas.org/ENSG00000032444-PNPLA6/tissue, including various brain regions, retina, peripheral nerves, liver, spleen, prostate, and placenta. Its expression pattern in mice shifts from widespread in early development to becoming restricted to specific neuronal populations as they mature [136–140], while in *Drosophila*, NTE/SWS is widely expressed throughout various tissues, with particularly high levels observed in the brain [141], including mushroom body and BBB cells [142,143]. Human PNPLA6/NTE gene is located on chromosome 19p13.2. Mutations in the PNPLA6/NTE gene have been linked to various neurodegenerative diseases, such as SPG39, Gordon-Holmes syndrome, Boucher-Neuhauser syndrome, Oliver-McFarlane syndrome, and Laurence-Moon syndrome, that are characterized by cerebellar ataxia, hypogonadotropic hypogonadism, chorioretinal dystrophy, spastic paraplegia, muscle wasting, peripheral neuropathy, and cognitive impairment [144]. Unfortunately, nowadays, there is no specific cure for these disorders. In humans, four canonical isoforms of PNPLA6/NTE generated by alternative splicing have been described. The canonical isoform 4, also known as NCBI isoform-a, contains 1375 amino acids. The PNPLA6/NTE enzyme is a lysophospholipase localized to the ER. PNPLA6/NTE is localized to ER and functions as a phospholipase B, hydrolyzing PC to GPC and two fatty acids via sequential PLA1 or PLA2 and lysophospholipase activities [145,146]. Mouse and human PNPLA6/NTE share an impressive 96 % identity in their amino acid sequences [138].

PNPLA7/NRE exhibits high expression in all tissues characterized by active energy metabolism and lipid turnover, including brain [147], with its levels being modulated by nutritional status https://www.proteinatlas.org/ENSG00000130653-PNPLA7/tissue. Compared to PNPLA6/NTE, the molecular and physiological functions of the closely related PNPLA7/NRE are less well characterized. PNPLA7/NRE, known as NTE-related esterase (NRE), is present in both the endoplasmic reticulum and LDs, together forming a distinct phylogenetic subfamily within PNPLAs [148]. The PNPLA7/NRE gene is located on human chromosome 9q34.3. Loss of PNPLA7/NRE leads to disruption in choline/methionine metabolism [106,149]. Recently, it was shown that PNPLA7 facilitates the conversion of LPC to GPC within this metabolic pathway. This process promotes the production of free choline, contributing methyl groups to the methionine cycle [106]. The canonical isoform of PNPLA7/NRE, also known as NCBI isoform-b, contains 1317 amino acids. PNPLA7/NRE, functioning as a lysophospholipase, exhibits a preference for hydrolyzing unsaturated forms of LPC, a significant signaling molecule and constituent of cellular membranes, resulting in the generation of GPC and a FFA [148]. GPC serves as a precursor to endogenous choline, with its methyl groups being primarily directed into the methionine cycle within the liver [106,148,149]. PNPLA7/NRE is localized to the ER and LDs, where its N-terminal region is anchored to the ER and the C-domain associates with LDs [150,151]. Interestingly, cyclic nucleotides can regulate the binding of PNPLA7/NRE to LDs in fat storage organelles [152].

Overall, loss of the lysophospholipases PNPLA6/NTE or PNPLA7/NRE results in dysregulation of lipid metabolism, characterized by elevated levels of PC, LPC, and LPA and a decreased level of GPC [59,66,106,139,151,153].

## 5. The functional impact of PNPLA6/NTE or PNPLA7/NRE knockout models on developmental defects, metabolic dysregulation, and disease mechanisms

Complete PNPLA6/NTE knockout in mice leads to embryonic lethality due to placental development defects, absence of placental labyrinth formation, and breakdown of yolk sac circulation, leading to enlarged pericardia, and dilated blood vessels in the embryo [154]. Importantly, heterozygous PNPLA6/NTE knock-out animals develop normally, yet show reduced phospholipase activity and increased susceptibility to certain OPs [155]. Conditional knockouts in mice brains, using Nes-cre, result in motor coordination defects and neuronal degeneration in aged animals, including Purkinje cell loss, ER disruptions, and abnormal reticular aggregates [156]. Importantly, glial-specific knock-outs, using GFAP-cre, exhibited incomplete ensheathment of Remak fibers in the sciatic nerve [140], while a selective testis knock-down implicated PNPLA6/NTE in spermatogonial stem cell proliferation, with reduced sperm count observed in OP-treated male mice [140,157]. Silencing PNPLA6/NTE in a human pluripotent cell line induced similar changes in the transcriptome related to neuronal development, respiratory and vascular system formation, suggesting that the function of PNPLA6/NTE during development is conserved in humans [158,159].

Furthermore, impaired myelination in both the central and peripheral nervous systems upon PNPLA6/NTE and certain LSDs, such as Krabbe’s disease [140,160], demonstrate possible connection between these suggesting importance of PNPLA6/NTE lysosomal function for vertebrate myelination. Interestingly, it has been shown that hereditary SPG15 leads to lysosomal swelling, strengthening the link between hereditary spastic paraplegia and lysosomal dysfunction [161]. However, the relationship between lysosomal abnormalities and neuronal death remains insufficiently explored. The treatment approach for SPG39 focuses on symptom management through physical therapy, muscle relaxants, and pain medications, as there is currently no specific cure and treatment is primarily supportive. Similarly, rare genetic disorders such as Gordon-Holmes syndrome, Boucher-Neuhauser syndrome, Oliver-McFarlane syndrome, and Laurence-Moon syndrome are managed symptomatically, often requiring a multidisciplinary approach involving medications, physical and occupational therapy, and other supportive interventions.

Complete PNPLA7/NRE knockout mice are born normally but experience lower body temperature, muscle weakness, reduced visceral and subcutaneous fats, and die within a few months. Moreover, PNPLA7/NRE loss leads to smaller LDs, more mitochondria, and features of beige adipocytes with increased thermogenic marker expression [106]. Metabolome analysis shows reduced levels of GPC, choline, and phosphocholine, with elevated LPC species, indicating role of PNPLA7/NRE as a major lysophospholipase hydrolyzing LPC to GPC in the liver [106]. Interestingly, PNPLA7/NRE was found to be highly expressed in naïve macrophages. Moreover, knockdown of PNPLA7/NRE increased the inflammatory gene expression in lipopolysaccharide (LPS)-challenged macrophages. PNPLA7/NRE is expressed in macrophages and downregulated during LPS-induced M1 polarization, where it acts as a suppressor of proinflammatory responses, likely through its lysophospholipase activity on LPC and modulation of intracellular LPC metabolism. [162]. Interestingly, PNPLA7/NRE has been shown to play a role in tumorigenesis [134]. Moreover, PNPLA7/NRE is significantly downregulated in gastric and colorectal cancer tumor tissues, and HCC [134,149,163]. Given that insulin downregulates PNPLA7/NRE expression [150], and considering the constitutive activation of insulin signaling in HCC, it is plausible that insulin signaling in liver cancer could lead to the suppression of PNPLA7/NRE expression [149]. Moreover, hypermethylation leads to reduced expression levels of PNPLA7/NRE in HCC [163]. Deficiency of PNPLA7/NRE in the liver has been demonstrated to decrease the secretion of very-low-density lipoproteins, indicating that PNPLA7/NRE potentially stabilizes ApoE through protein-protein interactions, irrespective of its lysophospholipase activity [134,164]. Considering that ApoE is a significant genetic risk factor for late-onset Alzheimer’s disease, often associated with neuroinflammation, PNPLA7/NRE might also play a role in the development of neurological conditions. Importantly, PNPLA7/NRE loss reduces mTOR pathway activity in human skeletal muscle cells. Additionally, insulin can suppress PNPLA7/NRE expression, suggesting its involvement in skeletal muscle energy metabolism [148]. Together, PNPLA6/NTE and PNPLA7/NRE knockout studies highlight the critical roles of these enzymes play in development, neuronal function, and metabolic regulation.

## 6. *Drosophila* NTE/SWS: Homology to PNPLA6 and PNPLA7 and its impact on neurodegeneration, lipid metabolism, and cellular stress

*Drosophila melanogaster* serves as an excellent genetic model for investigating various human diseases, overcoming challenges like extended lifespan, ethical concerns, and limited controls in human studies. Fruit fly is a great model for investigating the molecular mechanisms of age-dependent neurodegenerative diseases, providing valuable insights into disease pathology and drug targets, while also avoiding ethical concerns associated with human research [165,166].

The *Drosophila* Swiss Cheese (NTE/SWS) protein, functioning as a highly conserved lysophospholipase, exhibits a 39 % amino acid sequence homology with mouse PNPLA6/NTE [138]. Moreover, *Drosophila* NTE/SWS shares high conservation with human PNPLA7/NRE, exhibiting 65 % amino acid sequence homology. Similar to its human orthologues, the *Drosophila* NTE/SWS protein exhibits the presence of four distinct domains: TM domain; 3 cNMP domains; highly conserved phospholipase domain, and a fourth domain capable of binding to the PKA-C3 [167]. Importantly, analysis and comparison of the predicted structures of phospholipase domains of human PNPLA6/NTE and *Drosophila* NTE/SWS demonstrates their overlapping nature. In both proteins, the phospholipase domains show a high degree of confidence as helices, with predicted local distance difference test scores (pLDDT) exceeding 90 [143], suggesting a high level of accuracy and reliability in their structural predictions.

Initially, mutations in the *swiss cheese* (*sws*) gene were linked to neurodegeneration, causing brain vacuoles, earning the name “swiss cheese”, and impaired locomotion in flies. Null *sws*^*1*^ mutation leads to age-dependent neuronal degeneration, reduced lifespan, and locomotion deficits [141]. NTE/SWS is expressed in both neurons and glia, and is required in neurons and glia in a cell autonomous manner [168]. Neuronal *sws* knockdown leads to neurodegeneration, reduced longevity, and impaired locomotion [142]. Moreover, NTE/SWS loss results in abnormal mushroom body development, neuropile responsible for the memory formation, and memory defects [142]. Mutations in *sws* affect neuromuscular junction morphology and synaptic marker distribution. Glial *sws* knockdown highlights the essential role of NTE/SWS in ensheathing and surface glia, with BBB-specific downregulation resulting in vacuoles, impaired locomotion, and gaps in the BBB [143,169,170].

**Fig. 3.**
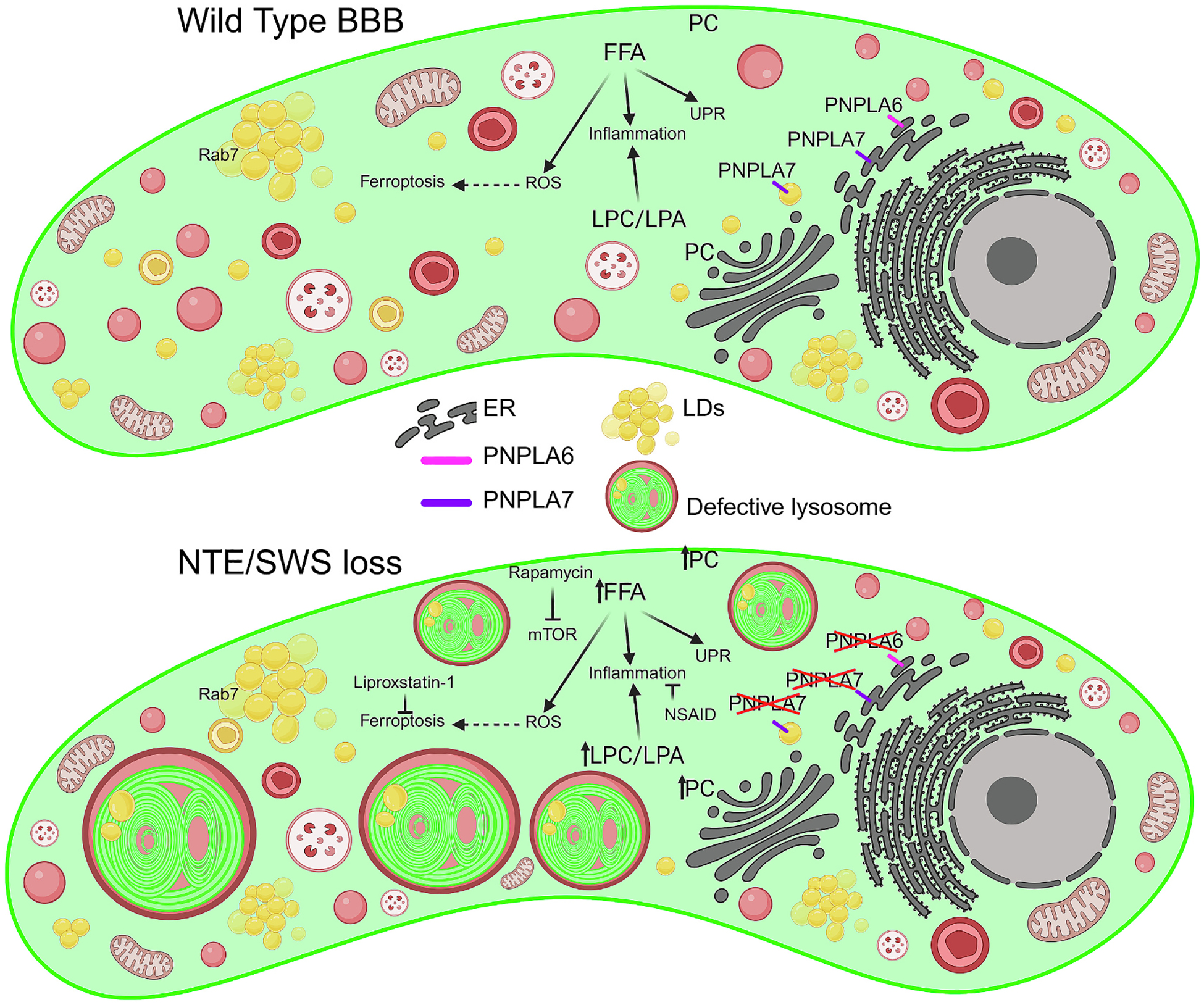
*Drosophila* NTE/SWS: Homology to PNPLA6 and PNPLA7 and its impact on neurodegeneration, lipid metabolism, and cellular stress. The schematic illustrates the localization of PNPLA6/NTE and PNPLA7/NRE homolog (NTE/SWS) and its involvement in various cellular processes, including lipid droplet biogenesis, endosomal-lysosomal function, inflammatory response, ER stress, ferroptosis, and oxidative stress. PNPLA6/NTE localizes to the ER where it catalyzes the hydrolysis of PC into LPC or GPC, generating FFA. This process plays a role in maintaining lipid homeostasis, supporting cellular membrane integrity, modulating inflammatory responses, regulating ER homeostasis, controlling ROS levels, and ensuring proper mitochondrial function. PNPLA7/NRE localization to the ER and LDs, suggests its role in the LDs formation and function. Proper levels of NTE/SWS are essential for maintaining the structural integrity and functional stability of the BBB. Loss of NTE/SWS results in elevated levels of PC, LPC, LPA and FFAs. PC is a major structural phospholipid in cellular membranes, and its accumulation may disrupt membrane architecture and lipid raft organization. LPC, as well as LPA, is known to mediate pro-inflammatory responses in various disease states, contributes to heightened inflammation when present at elevated levels. FFAs, in addition to their role in membrane composition, are key mediators in several signaling pathways. Their excess can activate the mTOR pathway, induce ER stress, promote apoptosis and ferroptosis, and contribute to insulin resistance. The accumulation of PC, LPC, and FFAs might lead to increased LD formation. Impaired LD biogenesis results in uncontrolled lipolysis of existing LDs, triggering ER stress and activating the UPR. This cascade is further marked by increased ROS, neuronal mitochondrial dysfunction, accumulation of Rab7-positive vesicles, and the formation of enlarged, dysfunctional lysosomes. Importantly, treatment with NSAIDs, rapamycin, or liproxstatin-1 in NTE/SWS-deficient flies has shown potential in restoring BBB integrity. Created in BioRender. Tsap, M. (2025) https://BioRender.com/j39h702

The *Drosophila* NTE/SWS is a highly conserved lysophospholipase, that regulates PC metabolism, thus influences lipid homeostasis. NTE/SWS loss in fruit fly leads to elevated levels of PC, LPC and LPA [168,171]. Moreover, the complete loss of NTE/SWS, or its downregulation in neurons or glia, results in the accumulation of LDs [142]. Interestingly, PNPLA7/NRE is localized to LDs [150,151], while mammalian PNPLA6/NTE is not localized there despite its intrinsic affinity for the LD surface (Fig. 3) [142,172,173]. Lipids, such as fatty acids, are essential for cellular functions, but its deregulation can lead to toxicity and cell death. LDs, once seen as simple fat inclusions, are now recognized as dynamic organelles crucial for lipid and energy homeostasis, regulating the distribution of PUFAs [43]. Our recent data indicate that the loss of NTE/SWS leads to elevated levels of both saturated and unsaturated FFAs [143], suggesting that PNPLA6/NTE is crucial for proper fatty acid metabolism.

LDs, formed in the ER, are dynamic organelles that associate with various cellular structures such as the ER, Golgi, mitochondria, peroxisomes, and lysosomes at specialized locations called membrane contact sites (MCSs) to exchange lipids and coordinate fatty acid storage and release [48]. Impaired LD biogenesis causes lipolytic release of FAs from the existing LDs, leading to ER stress (Fig. 3) and the activation of the unfolded protein response (UPR) [43]. The mechanism linking impaired LD biogenesis to UPR activation is unclear, but it may involve aberrant fatty acid storage disrupting ER homeostasis, protein folding, or calcium storage, or it could directly alter the ER membrane lipid composition. Due to the recent studies, changes in ER lipid composition can directly activate the UPR [43,174,175].

Interestingly, NTE/SWS influences ER stress responses through alterations in membrane lipid composition, with its loss leading to elevated levels of the chaperone GRP78 and increased XBP1 splicing [176]. ER stress responses can also be triggered by changes in lipid composition that inhibit the Sarco-Endoplasmic Reticulum Ca2 + -ATPase (SERCA), leading to a reduction in ER Ca2+ stores and activation of the UPR [177]. Importantly, NTE/SWS deficiency leads to reduced SERCA levels [176].

Another study demonstrates that elevated ROS levels and neuronal mitochondrial dysfunction lead to the accumulation of LDs in glia in *Drosophila* [46]. Moreover, in neuropathological conditions like amyotrophic lateral sclerosis (ALS), levels of fatty acid β-oxidation are upregulated, as well as levels of oxidative stress mediated by ROS [178].

NTE/SWS loss leads to an abnormal mitochondrial signal in the mushroom bodies, accompanied by an increase in the total number of mitochondria and a decrease in the number of mobile mitochondria in the fly brain [142]. Analysis of ROS levels showed increased acceleration in flies with NTE/SWS deficiency (Fig. 3). Expression of human PNPLA6/NTE alleviated ROS levels in loss-of-function mutants, suggesting a connection between NTE/SWS downregulation and oxidative stress. Moreover, the upregulation of antioxidant defense genes in flies with neuronal NTE/SWS loss implies that oxidative stress results from NTE/SWS downregulation [142,169].

Our recent study shows that NTE/SWS-associated neurodegeneration involves dysfunctional lysosomes [143]. Furthermore, considering that Rab7 facilitates LD trafficking to lysosomes for degradation, and that NTE/SWS loss results in excessive LD and Rab7-positive accumulations [143], these findings suggest that PNPLA7/NRE localization to LDs may be involved in endosomal-lysosomal function. Understanding the shared mechanisms between PNPLA6/NTE-associated neurodegeneration and LSDs could reveal common pathways in disease progression, potentially leading to repurposed treatments for LSDs or new therapies for PNPLA6/NTE-linked conditions.

Interestingly, NTE/SWS plays a crucial and evolutionarily conserved role in maintaining the structure and permeability of the BBB, as well as in regulating the organization of plasma membrane domains and TJ rafts [143]. The loss of NTE/SWS disrupts BBB integrity, but this can be partially mitigated by treatment with sodium salicylate, a non-steroidal anti-inflammatory drug (NSAID), indicating that an activated inflammatory response is associated with PNPLA6/NTE deficits (Fig. 3). *Drosophila* peroxidase enzyme (Pxt), potentially an insect cyclooxygenase orthologue, could be targeted by NSAID treatment [179–181]. Additionally, rapamycin, which activates autophagy by inhibiting mTOR, can also help restore BBB function. Neurodegenerative disorders like AD, PD, Huntington disease (HD), and ALS are associated with BBB dysfunction, especially in advanced stages [182]. Our study identified that neurodegeneration associated with NTE/SWS is linked to BBB breakdown, leading to upregulation of FFA levels and progressive neuroinflammation, which is linked to dysfunctional lysosomes. While the extent of neuroinflammation and the specific molecular pathways involved may vary across different diseases, these findings suggest a broader role of lysosomal dysfunction in neuroinflammation. Unsaturated FFAs like palmitoleic, oleic, and linoleic acids contribute to oxidative stress and lipotoxicity, triggering ER stress, calcium imbalance, mitochondrial dysfunction, and cell death [183–185].

Moreover, saturated FFAs activate mTOR pathway and induce cell apoptosis [186]. PUFAs are precursors of lipid mediators that regulate inflammation and are linked to Alzheimer’s disease and dementia [187,188]. Furthermore, elevated levels of PUFAs and ROS can induce ferroptosis, a form of cell death associated with lipid hydroperoxides formed through the oxidation of PUFAs. It has been shown that BBB dysfunction is associated with lipid peroxidation and iron accumulation, which impact TJ integrity. Investigating the connection between ferroptosis and BBB dysfunction may offer new therapeutic opportunities for brain disorders [189]. Ferroptosis is characterized by distinct morphological, biochemical, and genetic features that differentiate it from other cell death forms such as necrosis and apoptosis [190]. Notably, the ferroptosis inhibitor liproxstatin-1 has shown potential in mitigating compromised BBB integrity in NTE/SWS-deficient models (Fig. 3), suggesting that NTE/SWS-associated neurodegeneration may involve ferroptotic cell death [143].

Additionally, lysosomal activities have a direct impact on immunity. In innate immunity, pathogens like bacteria are engulfed through phagocytosis and directed to lysosomes for breakdown. If bacteria evade phagosomes and enter the cytosol, autophagy provides an extra defense by capturing and transporting them to lysosomes for degradation. Furthermore, the transport of cholesterol from lysosomes to the ER is inhibited when mTOR is activated. This mTOR activation disrupts lysosomal function [191]. Moreover, lipid accumulation triggers the activation of mTORC1, which is associated with the process of lipogenesis [192]. Our research suggests that administering rapamycin treatment can alleviate NTE/SWS-associated symptoms (Fig. 3), indicating that mTOR-related lipid imbalance contributes to progressive neurodegeneration [143].

Recent transcriptome analyses have revealed distinct patterns of gene upregulation in both glia and neurons following NTE/SWS loss. Neuronal NTE/SWS knockdown resulted in the identification of 589 upregulated genes, with notable enrichment in processes such as phototransduction, visual perception, amino acid and lipid metabolism, defense response, and GPCR signaling. Additionally, 77 of these genes were associated with oxidation-reduction processes, including 12 involved in the major intracellular antioxidant system, suggesting a role in glutathione metabolism [142]. In contrast, NTE/SWS loss in BBB cells led to the upregulation of 856 genes associated with infection response, immunity, proteolysis, and metabolism of carboxylic acids and glutathione [169].

The high conservation of the NTE/SWS protein between *Drosophila* and humans highlights its significance in understanding disease mechanisms and identifying therapeutic targets. Moreover, exploring the role of NTE/SWS in regulating lysosomal function and its impact on neuroinflammation and oxidative stress could provide further insights into potential treatments for neurodegenerative diseases. Given the potential for repurposing existing drugs, such as NSAIDs and rapamycin, and the development of novel ferroptosis inhibitors, there is promising potential for translating these findings into therapeutic strategies for human conditions. Continued use of *Drosophila* models in combination with advanced genetic, biochemical, and pharmacological approaches will be crucial for advancing our understanding and treatment of these debilitating diseases.

## 7. Future directions

Systemic inflammation is a hallmark of many age-related disorders. While aging is commonly associated with chronic, low-grade inflammation (inflammaging), it is now well-established that neuroinflammation can occur independently within the CNS, despite the brain’s immune-privileged status and the protective role of the BBB. Emerging evidence suggests that shared inflammatory mechanisms underlie a broad spectrum of neurodegenerative diseases, highlighting opportunities to identify common therapeutic targets.

Recent findings, including those discussed in this review, point to a role for PNPLA family members in modulating inflammatory responses. For example, PNPLA2 supports endothelial barrier function and vascular homeostasis by converting LD-derived AA into PGI2, generating both pro- and anti-inflammatory lipid mediators via the cyclooxygenase and lipoxygenase pathways depending on the cellular context. PNPLA8 produces inflammatory signaling lipids such as 2-AA-LPC and mitochondrial eicosanoids; its loss reduces eicosanoid production, disrupts mitochondrial function, and promotes oxidative stress. Similarly, PNPLA9 protects against lipid peroxidation and ferroptosis, while its deficiency increases mitochondrial lipid peroxidation and ROS production, contributing to neurodegenerative phenotypes such as parkinsonism, microcephaly, and axonal dystrophy.

In addition to roles in inflammation, several PNPLAs are implicated in neurodevelopment. Loss of PNPLA6 or PNPLA8 results in progressive neurodegenerative phenotypes, including cerebellar ataxia, peripheral neuropathy, and microcephaly, accompanied by elevated oxidative stress and lipid peroxidation. Mutations in PNPLA9 are linked to infantile neuroaxonal dystrophy and early-onset Parkinsonian syndromes, underscoring the clinical relevance of this enzyme family in maintaining neuronal integrity and regulating inflammation. Moreover, PNPLA7/NRE acts as a suppressor of pro-inflammatory responses in macrophages by metabolizing LPC. Its knockdown enhances inflammatory gene expression, and it is downregulated in various cancers and insulin-resistant conditions, linking lipid metabolism, inflammation, and neurodegeneration. These proteins share structural and functional similarities and are involved in lipid metabolism and inflammatory signaling. Many PNPLAs localize to the cytosol, organelle membranes, or LDs—dynamic structures essential for energy storage, lipid signaling, and cellular stress responses. Their close association with LDs suggests that PNPLAs are key modulators of LD-associated cellular processes. Among them, PNPLA6 and PNPLA7 have emerged as particularly important regulators of lipid homeostasis.

Studies in various models have shown that loss of PNPLA6/NTE leads to abnormal lipid metabolism and plays a critical role in maintaining various cellular processes. *Drosophila* NTE/SWS, a highly conserved protein, shares 39 % amino acid identity with mouse PNPLA6/NTE and 65 % with human PNPLA7/NRE. In *Drosophila*, loss of NTE/SWS results in upregulated phospholipids and the accumulation of PC and LPC, leading to excessive membrane production, disrupting the lipid composition of the BBB, altering lipid rafts and impairing signaling by preventing efficient interactions among key components. Lipid rafts, enriched with specific lipids such as sphingolipids and cholesterol, create microdomains in the plasma membrane that cluster signaling receptors, enzymes, and other molecules.

**Fig. 4.**
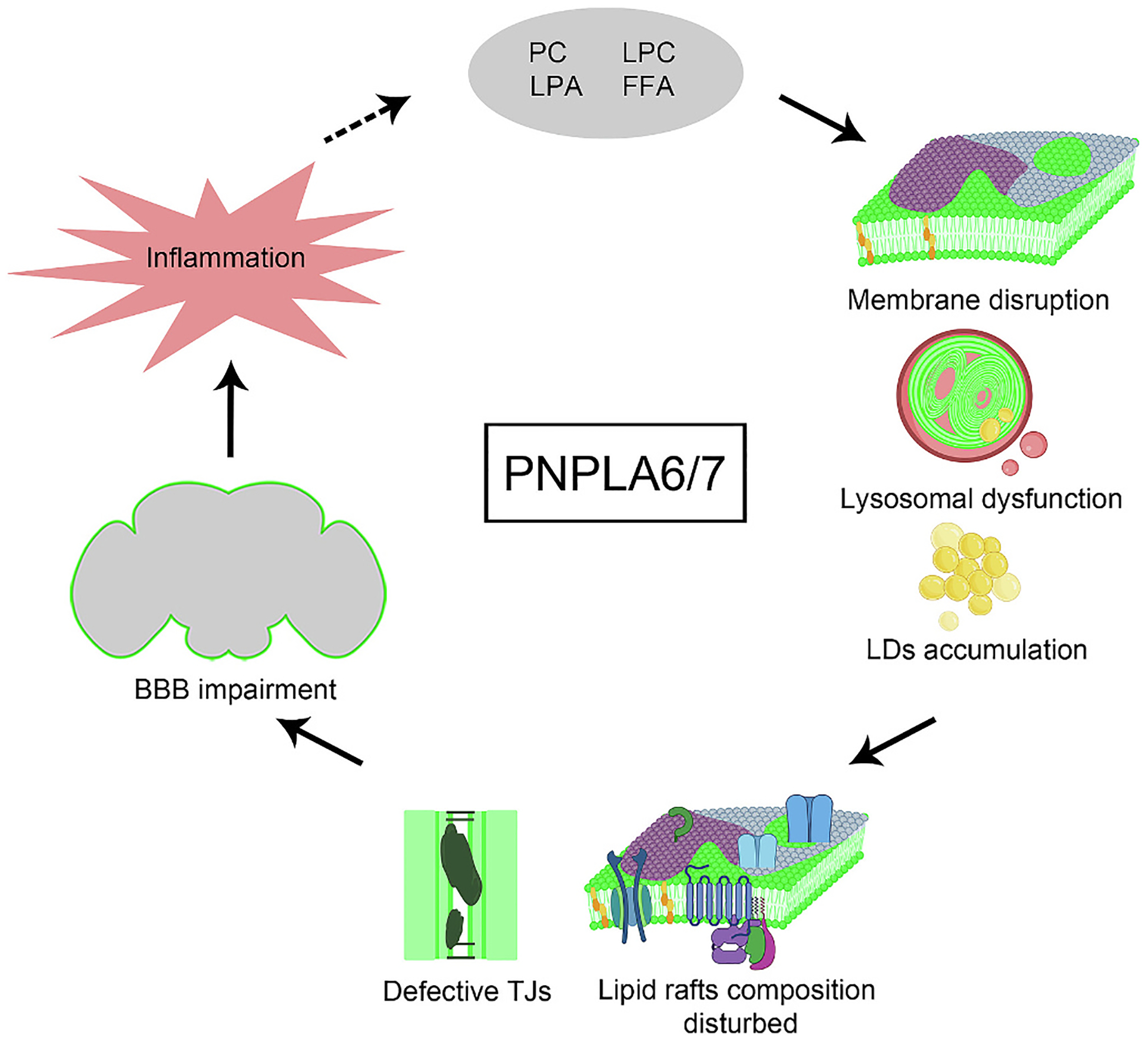
The loss of NTE/SWS, PNPLA6/7 orthologue, results in elevated levels of PC, LPC, LPA, and FFAs that disrupts lipid membrane homeostasis, resulting in excessive membrane formation, lipid droplet accumulation, and lysosomal abnormalities. As the consequence lipid rafts, including TJs are disrupted leading to BBB impairment and inflammation. Created in BioRender. Tsap, M. (2025) https://BioRender.com/e71j408 and Adobe Photoshop software. Figures were created using Adobe Photoshop software, AlphaFold3 [193], PyMOL Molecular Graphics System, v2.5.5 [Schrödinger, LLC; https://pymol.org/], and BioRender.

This phospholipid imbalance overwhelms normal lipid processing pathways, and lysosomal membranes—sensitive to changes in lipid composition—are particularly affected. Lysosomal function depends on a precise lipid balance to maintain acidity and enzymatic activity. Disruption due to NTE/SWS deficiency impairs not only lysosomal degradation but also critical signaling pathways, as lysosomes serve as hubs for nutrient sensing and cellular stress responses. NTE/SWS thereby influences cellular signaling by maintaining lipid raft and membrane integrity; its loss causes imbalanced lipid homeostasis, excess membrane formation, LD accumulation, and lysosomal dysfunction. Furthermore, NTE/SWS deficiency leads to FFA imbalance and disrupted ER homeostasis, triggering ER stress and activation of the UPR. Saturated FFAs contribute to mTOR activation, ER calcium imbalance, and ferroptosis. Inhibition of ferroptosis (e.g., with liproxstatin-1) alleviates symptoms. Additionally, anti-inflammatory agents like sodium salicylate and rapamycin can partially restore BBB function, suggesting opportunities for repurposed therapeutics (Fig. 4). The potential role of LPC and LPA, which are affected by the loss of PNPLA6/NTE, warrants further investigation. As bioactive lipids, both LPC and LPA are involved in regulating various cellular processes, including inflammation. Given PNPLA6/NTE’s critical role in nervous system health, exploring how neuroinflammation might be modulated by LPC and LPA could be an important area of study.

Together, these data demonstrate the importance of lipid regulation and membrane organization for signaling, intracellular trafficking, and the overall health of key cellular compartments, including lysosomes. Future research should explore PNPLAs, in particular PNPLA6/NTE, function in lipid metabolism and membrane dynamics across three key areas: [1] how its loss disrupts lipid rafts and signaling, [2] the connection between NTE/SWS-related lysosomal dysfunction and diseases such as neurodegeneration and cancer, and [3] therapeutic strategies aimed at restoring lipid balance and membrane integrity.

## Figures and Tables

**Table 1:** Functional overview of PNPLA family members.

PNPLA member	Other Names	Localization	Substrate	Tissues	Functions	Diseases
PNPLA1	N/A	Cytoplasm, LDs	TAG and omega-hydroxyceramide	Epidermal keratinocytes	omega-O-acylceramide synthesis for skin barrier function	ARCI
PNPLA2	ATGL	Cytoplasm, LDs	TAGs	Ubiquitous, highly expressed in adipose tissue and heart	TAG hydrolysis, Lipolysis, Eicosanoid generation	Neutral lipid storage disease with myopathy
PNPLA3	N/A	Cytoplasm, LDs	TAGs	Adipose, liver	Hepatic fat content regulation	MAFLD, MASH
PNPLA4	GS2	Cytoplasm	TAGs, REs	Ubiquitous, keratinocytes, adipose, liver, muscle, heart Absent in mice	Retinoic acid levels regulation	Unknown
PNPLA5	GS2-like	Cytoplasm	TAGs,	Ubiquitous, low expression in many tissues, but adipose, lung, brain, pituitary	Lipid metabolism	Unknown
PNPLA6	NTE	ER	PC, LPC	Ubiquitous, especially nervous system	PC metabolism, nervous system integrity	SPG39, Gordon-Holmes syndrome, Boucher-Neuhauser syndrome, Oliver-McFarlane syndrome, and Laurence-Moon syndrome
PNPLA7	NRE	ER, LDs	LPC	Ubiquitous, especially liver, adipose, pancreas, muscle,	LPC metabolism, energy metabolism regulation	Disturbed choline/methionine metabolism
PNPLA8	iPLA2γ	ER, mitochondria, peroxisome, autophagosome	Phospholipids	Ubiquitous, heart, nervous system, adipose	2-AA-LPC production, Deduces hepatic steatosis, Mitochondrial bioenergetics modulation	Neurodegeneration, adipose tissue atrophy, myopathy
PNPLA9	iPLA2β, PLA2G6	Cytoplasm, binding to various organelle membranes	Phospholipids	Ubiquitous, especially testes, nervous system, pancreatic islets	Protection from ferroptosis by removing oxidized phospholipids, AA production, Mitochondrial bioenergetics modulation, Acrosome Exocytosis, Insulin Secretion, Apoptosis, Autophagy, Modulating Ion Channels, Axon Maintenance, Mitochondrial Repair	Neurodegeneration, metabolic disease, infertility
